# The Role of Immune Checkpoint Blockade in Uveal Melanoma

**DOI:** 10.3390/ijms21030879

**Published:** 2020-01-29

**Authors:** Anja Wessely, Theresa Steeb, Michael Erdmann, Lucie Heinzerling, Julio Vera, Max Schlaak, Carola Berking, Markus Vincent Heppt

**Affiliations:** 1 Department of Dermatology, Universitätsklinikum Erlangen, Friedrich Alexander University, Ulmenweg 18, 91054 Erlangen, Germany; anja.wessely@uk-erlangen.de (A.W.); theresa.steeb@uk-erlangen.de (T.S.); michael.erdmann@uk-erlangen.de (M.E.); lucie.heinzerling@uk-erlangen.de (L.H.); julio.vera-gonzalez@uk-erlangen.de (J.V.); carola.berking@uk-erlangen.de (C.B.); 2 Department of Dermatology and Allergy, University Hospital, LMU Munich, Frauenlobstr. 9-11, 80337 Munich, Germany; max.schlaak@med.uni-muenchen.de

**Keywords:** uveal melanoma, ocular melanoma, immune checkpoint blockade, CTLA-4, cytotoxic T lymphocyte-associated antigen, ipilimumab, PD-1, programmed death 1, pembrolizumab, nivolumab

## Abstract

Uveal melanoma (UM) represents the most common intraocular malignancy in adults and accounts for about 5% of all melanomas. Primary disease can be effectively controlled by several local therapy options, but UM has a high potential for metastatic spread, especially to the liver. Despite its clinical and genetic heterogeneity, therapy of metastatic UM has largely been adopted from cutaneous melanoma (CM) with discouraging results until now. The introduction of antibodies targeting CTLA-4 and PD-1 for immune checkpoint blockade (ICB) has revolutionized the field of cancer therapy and has achieved pioneering results in metastatic CM. Thus, expectations were high that patients with metastatic UM would also benefit from these new therapy options. This review provides a comprehensive and up-to-date overview on the role of ICB in UM. We give a summary of UM biology, its clinical features, and how it differs from CM. The results of several studies that have been investigating ICB in metastatic UM are presented. We discuss possible reasons for the lack of efficacy of ICB in UM compared to CM, highlight the pitfalls of ICB in this cancer entity, and explain why other immune-modulating therapies could still be an option for future UM therapies.

## 1. Introduction

Uveal melanoma (UM) represents the most common ocular malignancy in adults. About 85% to 90% of the primary tumors arise from melanocytes residing in the choroid, the pigmented layer of the eyeball that also contains blood vessels [1,2], and less frequently from the ciliary body or iris of the eye where melanocytes are also present [3]. With an incidence of 4–7 cases per million in Europe, it is much rarer than cutaneous melanoma (CM) [4]. Several factors have been associated with an increased risk of developing UM. These include the presence of choroidal or cutaneous nevi [5], fair skin, light eye color [6], oculodermal melanocytosis [7,8], inactivating mutations of the tumor-suppressor BRCA1-associated protein 1 (BAP1) [9,10], and exposure to ultraviolet (UV) radiation [11].

### 1.1. Cutaneous vs. Uveal Melanoma: Genetic Differences and Implications for Prognosis

Although UM and CM both arise from melanocytes and share similar risk factors, these melanoma subtypes substantially differ biologically and clinically [5]. Similar to other cancer entities that are associated with the exposure to environmental carcinogens such as nonsmall cell lung cancer (NSCLC), CM has an extremely high mutational burden with up to 100 mutations per megabase [12,13]. Despite this huge number, a few typical driver mutations are found in the majority of CM tissue samples that affect members of the BRAF-MEK-ERK signaling cascade. About 40% to 50% harbor mutations in the *BRAF* gene coding for v-Raf murine sarcoma viral oncogene homolog, and about 20% harbor mutations in the *NRAS* gene coding for neuroblastoma rat sarcoma viral oncogene homolog [14,15,16,17]. Both activating *BRAF* and *NRAS* mutations lead to a constitutive activation of the mitogen-activated protein kinase (MAPK) signaling pathway that promotes proliferation and survival, and thereby contribute to cancer formation and progression [18,19].

In contrast to CM, the number of mutations in UM is extremely low [20], and interestingly, typical CM driver mutations are not present in UM and vice versa. Instead of *BRAF* and *NRAS* mutations, which are almost never observed in UM [21,22], more than 80% of all UM harbor mutations in the genes encoding the guanine nucleotide-binding proteins Q polypeptide (GNAQ) and α11 (GNA11) [21,22]. *GNAQ* and the closely related *GNA11* encode Gα subunits of heterotrimeric G-proteins that interact with G-protein-coupled receptors. In about 90% of all cases, codon 209 [23] located in the Ras-like GTPase domain of the proteins is affected [24], and most commonly, glutamine is substituted by leucine (Q209L). This blocks the GTPase activity of the enzyme, resulting in a constantly bound GTP and thus a constitutive activation of the PLCβ/PKC pathway and downstream RAF-MEK-ERK signaling [21,22,25]. Besides, other downstream pathways as Trio-Rho-Rac and YAP-Hippo get activated by mutated Gα proteins [26]. A high PI3K-Akt-mTOR activity is also frequently observed in UM [27]; however, this seems to be the result of a phosphatase and tensin homolog (PTEN) expression loss [28], rather than due to mutated Gα proteins [28,29]. Other driver mutations in UM are by far less frequently detected and involve *CYSLTR2* encoding the G-protein-coupled cysteinyl leukotriene receptor 2 and *PLCB4* coding for phospholipase C β4, which act immediately upstream and downstream of GNAQ/11 in the signal transduction cascade [20,30,31].

Inactivating mutations in *BAP1* are present in about 40% to 47% of UM primary tumors and 80% of UM metastases [32]. BAP1 is a tumor suppressor involved in the repair of DNA double strand breaks [33], and about 8% of UM patients carry BAP1 germline mutations leading to a loss of function [34]. Mutations in genes coding for splicing factor 3B, subunit 1 (*SF3B1*), which is required for RNA splicing, and the eukaryotic translation initiation factor 1A, x-linked (*EIF1AX*), are present in 29% and 48% [35,36,37], respectively. Commonly observed chromosomal aberrations in primary UM include monosomy of chromosome 3 and loss of chromosome 1p, 6q, and 8p, as well as amplifications of chromosome 1q, 6p, and 8q [38,39,40,41,42]. These alterations also decisively affect the patient’s prognosis.

### 1.2. Uveal Melanoma: Therapy and Prognosis

Several treatment approaches for primary UM are suitable to achieve sufficient control over local tumor growth and in most cases even preserve the vision of the affected eye [43,44]. These options for the primary tumor involve brachytherapy, external beam radiotherapy, photon-based radiation, or surgical approaches like local resection and enucleation of the affected eye [44,45,46]. However, up to 50% of the patients develop distant metastases [47], and unlike CM, UM primarily metastasizes to the liver [48]. The reasons for this selective behavior have not been fully elucidated yet. An enrichment of local growth factors, chemokines, and adhesion molecules, a slow blood flow, and the presence of proangiogenic tumor-associated macrophages (TAMs) have been discussed as factors that facilitate the homing, extravasation, and establishment of UM metastases in the liver. The slow blood flow in the hepatic sinusoids, together with the expression of vascular cell adhesion molecule-1 (VCAM-1) on endothelial cells, can promote the trapping of circulating UM cells in the liver [49]. Furthermore, the hepatic microenvironment is rich in locally produced growth factors such as insulin-like growth factor-1 (IGF-1), hepatic growth factor (HGF), and chemokines—such as CXCL12, which may contribute to homing and metastasis formation [50] and the receptors CXCR4 and c-Met, which bind to CXCL12 and HGF, respectively—are widely expressed in UM cells [51,52]. Thus, this interaction of UM cells and the hepatic microenvironment likely contributes to the establishment of hepatic metastases as UM primary tumors expressing high levels of c-Met are associated with a higher risk for developing liver metastases [52].

Despite the tremendous advances in the therapy of advanced-stage CM in the last decade, which have been highlighted in several systematic reviews [53,54], the prognosis of metastatic UM has not changed and is still almost as poor as 30 years ago [55]. Five-year median overall survival (OS) rates for primary UM are around 80% [55]. However, these rates dramatically drop after diagnosis of metastatic disease to a median OS of about 13.4 months [56] and to a two-year OS of only 8% [46]. The presence of certain genetic aberrations has an impact on the clinical outcome and prognosis of UM in general [30,57]. A poor prognosis has been associated with monosomy 3 [58,59], mutations in the *BAP1* gene, which is also located on chromosome 3p21.3 [60], and chromosome 8q amplifications [61]. Metastatic spread of UM occurs more often in tumors harboring *GNA11* than *GNAQ* mutations [62,63], and *SF3B1* mutations are associated with an intermediate risk of metastases and the onset of late-occurring metastases [64]. Among these aberrations, monosomy 3 seems to be the strongest predictor for disease progression [58]. On the other hand, the presence of *EIFAX1* mutations [64] and chromosome 6p amplifications [59,61] are associated with a better prognosis. Besides, four molecularly different UM subsets can be defined, which are associated with different clinical outcomes [30].

In the metastatic stage, UM therapy has largely been adopted from CM. Once metastases are present, the disease course is often aggressive, and the prognosis remains dismal. A variety of local liver-directed treatment options have been investigated in clinical trials, but most of them did not result in a better survival in metastatic disease [65,66]. The frequently occurring *GNAQ* and *GNA11* mutations that lead to a constitutive activity of the MAPK signaling pathway [21,22] provided the rationale for the use of small molecule inhibitors targeting the downstream kinases. Several inhibitors targeting MEK have already been developed and with trametinib, cobimetinib, and binimetinib, and three of them have been approved for metastatic BRAF-mutated CM in combination with a BRAF inhibitor [67,68,69]. However, a recent systematic review showed that UM is little responsive to MEK inhibition regardless of the inhibiting agent and combination partner [70]. For example, the potentially promising combination of binimetinib with the PKC inhibitor sotrastaurin showed no clinical efficacy, but a high number of patients developed severe adverse events, resulting in the termination of the respective phase Ib/II clinical trial [71]. The MEK inhibitor selumetinib showed promising results in a phase II study compared to chemotherapy, with a response rate of 14% [72]. Thus, the expectations were high that similar results would be achieved in the SUMIT trial, a prospective double-blind phase III study investigating selumetinib plus dacarbazine versus dacarbazine alone [73]. Unfortunately, disappointing results were observed, as only 3% of the patients responded, and the progression-free survival (PFS) could not be significantly prolonged by the therapy. Additionally, metastatic UM patients who received the multikinase inhibitor sorafenib have shown a significantly longer median PFS compared to placebo in the randomized phase II STREAM study. However, the median OS was not different between both groups (13 months with sorafenib vs. 12.2 months with placebo) [74]. Other targeted treatment approaches have also shown no promising results, as recently reviewed in [75,76].

In contrast to MEK inhibitors, immune checkpoint blockade (ICB) with programmed death 1 (PD-1) inhibitors and the anti-cytotoxic T lymphocyte-associated antigen (CTLA-4) antibody ipilimumab has shown strong survival benefits in recent years in CM [77,78,79,80]. However, patients with UM have been widely excluded from most of the pivotal trials. Here, we provide a comprehensive and up-to-date overview of the role of ICB in UM.

## 2. Immune Checkpoints in Cancer Therapy

The immune response is a delicate process that needs to be balanced carefully in order to fulfill its function in the defense against pathogens on the one hand and to avoid massive damage of healthy tissue on the other hand. Several immune checkpoints help to control autoimmunity and maintain self-tolerance. Cytotoxic CD8+ T lymphocytes (CTLs) are able to identify and destroy infected cells as well as tumor cells by recognizing epitopes of antigens that are presented by human leukocyte antigen (HLA) class I molecules on the cell surface and thereby play an essential role in maintaining the integrity of the body [81].

**Figure 1:**
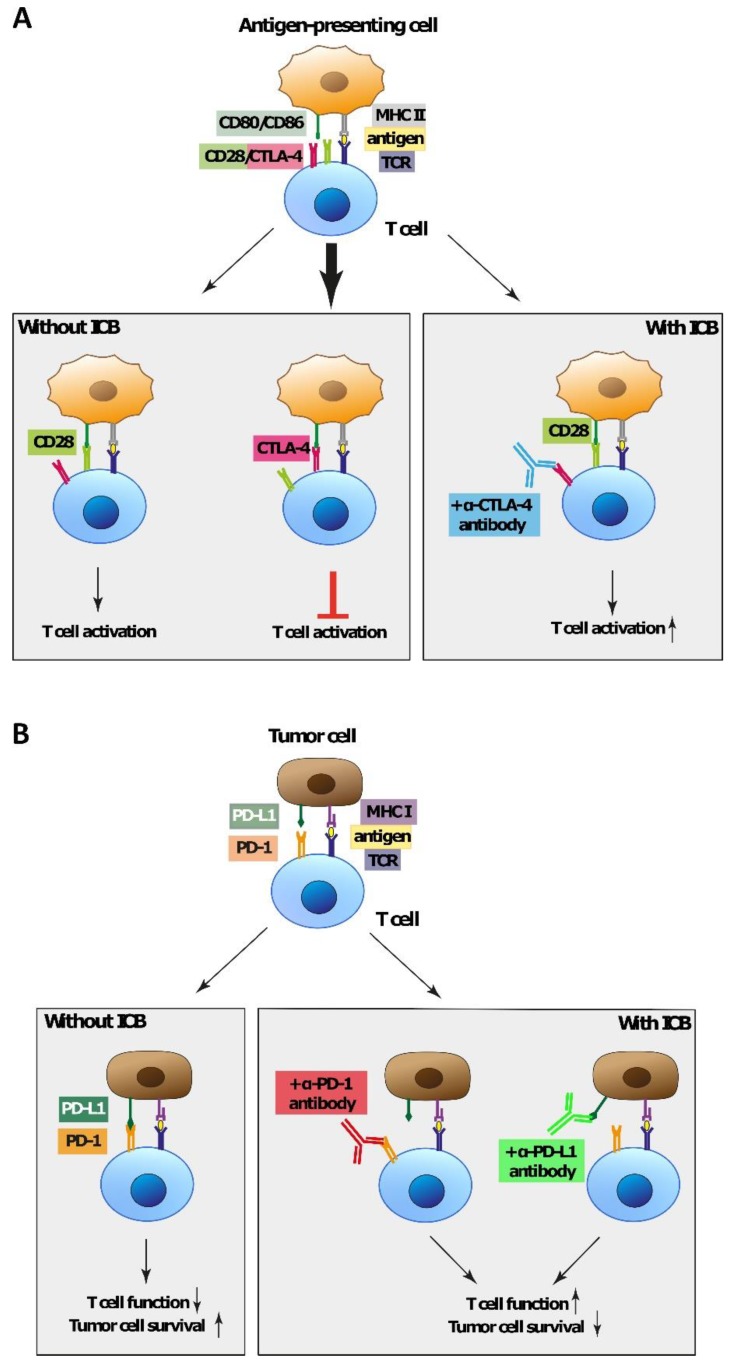
**Molecular mechanism of immune checkpoints**. (**a**) CTLA-4 is a critical negative regulator of the T cell response in the early activation phase of the adaptive immune response. It binds to the costimulatory ligands CD80 and CD86 on antigen-presenting cells with a higher affinity than CD28 and thereby prevents their interaction with CD28 and subsequent T cell activation. Anti-CTLA-4 antibodies block the interaction of CTLA-4 and CD80/86 and boost T cell activation and the anti-tumor response. (**b**) The PD-1–PD-L1 axis is an important mechanism to avoid tissue damage from autoreactive T cells and maintains the peripheral tolerance. Binding of PD-L1 to its receptor PD-1 blocks T cell receptor (TCR) signaling, resulting in limited T cell function. Antibodies targeting PD-1 or its ligand PD-L1 are able to inhibit their interaction and prevent the inactivation of tumor-reactive immune cells.

The anti-CTLA-4 monoclonal antibody ipilimumab was the first immune checkpoint inhibitor approved for therapy of metastatic CM by the US Food and Drug Administration (FDA) in 2011 [82]. CTLA-4 is a critical negative regulator of the T cell response. In general, T cell activation requires the transduction of two signals [83]: Signal 1 involves the interaction of a foreign peptide or mutated self-antigen bound to the major histocompatibility complex (MHC) on antigen-presenting cells (APCs) and the T cell receptor (TCR) on T cells. Additionally, a second antigen-independent signal has to be transduced by costimulatory B7 proteins CD80 (B7.1) and CD86 (B7.2) located on APCs that interact with the co-receptor CD28 on T cells [83]. Only both signals can fully activate T cells, which includes their clonal expansion and the expression of effector cytokines. CTLA-4 is expressed on both activated T cells and regulatory T cells (T_regs_). It is constitutively internalized; thus, about 90% of all CTLA-4 is found in intracellular vesicles unless its surface expression is upregulated due to stimulatory signals [84]. CTLA-4 binds to the costimulatory ligands CD80 and CD86 with a higher affinity than CD28 and thereby prevents their interaction with CD28 and subsequent T cell activation [85]. Additionally, this interaction also increases the expression of indoleamine 2,3-dioxygenase (IDO) in dendritic cells (DCs) [86]. IDO mediates immunosuppressive effects by converting the amino acid tryptophan to kynurenine, a metabolite that directly induces apoptosis in T cells. Furthermore, IDO-mediated tryptophan depletion impairs T cell function by leading to cell cycle arrest and anergy [87]. CTLA-4 binding to its ligands also leads to the removal of CD80/86 from the APC surface by a mechanism termed trans-endocytosis and further impairs T cell activation [88]. Thus, the number of activated T cells is controlled. CTLA-4 plays an important role in the maintenance of self-tolerance and the control of autoreactive T cells, as shown in studies with knockout mice [89]. The inhibitory effect of CTLA-4 can be overcome either by upregulating CD80/86 on APCs—which occurs physiologically in response to inflammatory stimuli in vivo—or therapeutically by administering antibodies targeting CTLA-4. These prevent the interaction with CD80/86 on APCs and additionally lead to the elimination of immunosuppressive T_regs_ via both direct cytotoxicity and antibody-dependent cell cytotoxicity in the tumor [85] and thereby boost T cell activation and the initiation of effector T cells (Figure 1A).

A second immune checkpoint that is exploited for cancer therapy involves the PD-1–PD-L1 axis. The interaction of the receptor PD-1 and its ligands programmed death ligand 1 (PD-L1) and programmed death ligand 2 (PD-L2) is an important mechanism to avoid tissue damage from autoreactive T cells and maintain the peripheral tolerance [90]. PD-L1 is constitutively expressed in low levels on the cell surface of nonhematopoietic cells as well as on T cells, B cells, macrophages, and DCs [90,91]. Mediated by proinflammatory cytokines such as interferon gamma (IFN-γ) and tumor necrosis factor-α (TNF-α) that are released by activated T cells, its expression is upregulated during inflammatory processes. PD-L1 binds to its receptor PD-1, which is widely expressed on T cells, B cells, and some myeloid cells [90]. When the TCR is simultaneously bound, the interaction of PD-L1 and PD-1 leads to the transduction of an inhibitory signal that blocks TCR signaling [91] by reducing PTEN phosphorylation, phosphoinositide 3-kinase (PI3K) inhibition [92], and MEK-ERK pathway activation [93]. This results in diminished T cell function due to reduced production of the T cell effector cytokines IFN-γ, TNF-α, and interleukin-2 (IL-2) and a decreased T cell proliferation and survival [94,95,96]. Thus, the PD-1–PD-L1 axis limits an extensive and too-long-lasting immune response that may damage healthy tissue. The second PD-1 ligand PD-L2 has been less extensively investigated yet. Its expression is limited to macrophages, DCs, mast cells, and B cells and can be induced by IL-4; however, the transcriptional regulation has not been fully elucidated yet [97]. In contrast to PD-L1, binding of PD-1 to PD-L2 suppresses the activation of T cells in lymphoid organs [98]. 

Tumor cells are capable of escaping T cells by exploiting the PD-1–PD-L1/PD-L2 axis to dampen the antitumoral immune response. PD-L1 can be expressed in high levels and on the majority of both tumor cells and tumor-infiltrating immune cells in several cancer entities like CM [77,99], NSCLC [100], epithelial ovarian cancer [101], and breast cancer [102]. Similarly, PD-L2 expression has also been detected in various tumor types on tumor cells, invading immune cells and endothelial cells [103]. Antibodies against PD-1 or its ligand PD-L1 are able to inhibit the PD-1-mediated transduction of inhibitory signals and prevent the inactivation of tumor-reactive immune cells. This strategy has been used successfully for the treatment of a variety of tumors including CM [77] (Figure 1B).

## 3. Studies Investigating Immune Checkpoint Blockade for UM Treatment

In the past, UM patients have been excluded from most trials investigating ICB for CM treatment. However, some prospective phase I and II trials have also included UM patients. An up-to-date summary of the most important pro- and retrospective analyses, dosage schemes, and study results are presented in the following overview (Table 1).

### 3.1. CTLA4-Blocking Antibodies

#### 3.1.1. Ipilimumab

The anti-CTLA-4 monoclonal antibody ipilimumab was the first immune checkpoint inhibitor approved for therapy of metastatic CM by the FDA in 2011 [82].

The prospective investigator-initiated SECIRA-UM trial investigated different dosages of ipilimumab combined with radiofrequency ablation (RFA) in 41 patients with UM and at least two liver metastases in a phase Ib/II trial [104]. No complete responses (CR) or partial responses (PR) were observed. Severe adverse events (AEs) were reported in 52% of the patients who had received the highest dosage (10 mg/kg), most frequently immune-related colitis. The frequency of grade 3 and 4 AEs was associated with a higher dosage of ipilimumab [104].

Five additional prospective studies with 185 patients in total investigated ipilimumab at a dose of 3 mg/kg body weight [105,106,107,108,109]. In the study published by Jung et al. [109], 10 UM patients (total *n* = 106 melanoma patients) were treated. The median PFS was 2.8 months (graphically determined based on the published time-to-event analyses), and it was unclear whether the median OS had been reached at the time of publication. Secondary outcomes were not reported; furthermore, the UM subgroup was poorly described [109]. In the other prospective studies that investigated this dosage scheme (3 mg/kg), overall response rates (ORR) ranged from 0% [108] to a sobering 4.8% [107]. The median PFS and OS were quite short, with a median PFS ranging from 2.8 months [108] to 3.6 months [107] and a median OS between 5.2 months [106] and 6.8 months [108]. The frequency of severe treatment-related AEs differed between the studies and ranged between 6% [107] and 36% [108].

Higher ipilimumab dosages of 10 mg/kg were investigated in three pro- and one retrospective analysis [110,111,112,113]. Interestingly, a prolonged median OS of up to 9.8 months was observed; however, the ORR did not differ from other trials investigating lower dosages. Median PFS was not determined in any of these four studies. Similar to the SECIRA-UM trial [104], the high dosage of ipilimumab was also associated with a higher number of severe AEs. Nine of the 10 study participants developed grade 3 AEs, and in one patient who participated in an adjuvant study, temporal arteritis resulted in persistent blindness [112]. 

Itchins et al. [114] reported the results of a single-center cohort analysis on 37 metastatic UM patients who received a sequential therapy with transarterial chemotherapy (TAC), ICB (predominantly ipilimumab), and a systemic chemotherapy. Although the ORR was comparably poor, this combined approach led to the longest median PFS (9 months) and OS (17 months) among all therapies investigating ipilimumab. However, interpreting these results may be problematic, as the authors did not distinguish between different ICB antibodies when reporting the outcomes [114]. 

Bol et al. [115] compared the efficacy of ipilimumab, pembrolizumab, and a combined therapy with ipilimumab and nivolumab in a retrospective population-based study including 86 patients. None of the 24 patients treated with ipilimumab responded to the therapy. The median PFS was 3.0 months and the median OS was 9.9 months [115]. 

#### 3.1.2. Tremelimumab

Tremelimumab (CP 675206) is another monoclonal CTLA-4-targeting antibody. In a multicenter phase II trial published by Joshua et al. [116], 11 UM patients received 15 mg/kg tremelimumab. None of the patients achieved an objective response (CR or PR). The median PFS was 2.9 and the median OS was 12.8 months. Secondary outcomes were not clearly reported. The poor results regarding PFS and radiological response led to the withdrawal of the trial at the first interim stage [116].

Taken together, the anti-CTLA-4 antibodies ipilimumab and tremelimumab showed a poor efficacy on the one hand with poor response rates and short PFS and OS, and its use in an adjuvant setting also achieved only discouraging results. On the other hand, a significant number of patients developed severe AEs. These results highlight the low efficacy and high toxicity of these antibodies for the treatment of metastatic UM. 

### 3.2. PD-1/PD-L1 Blocking Antibodies

#### 3.2.1. Nivolumab

The PD-1 blocking antibody nivolumab was evaluated in one prospective phase II trial [117] and three retrospective analyses [118,119,120].

In the prospective CheckMate 172 trial [117], a subgroup of 75 UM patients with progressive disease after anti-CTLA-4 therapy was included, 34 of whom were evaluable for outcome analysis. Two PR were achieved in this subgroup(ORR 6%), and 15 patients (44%) had stable disease (SD) after a minimum follow-up of 1 year. The median OS was 11 months, thus better than the median OS for ipilimumab in this group of patients. Data on the UM subgroup regarding PFS or severe AEs were not reported [117]. 

Three other reports retrospectively evaluated the efficacy of nivolumab in a rather low number of patients [118,119,120]. ORR ranged from 0% [118] up to 25% [119]. The median PFS was not reported in [119] and was 2.3 months in the other studies, and the median OS ranged from 9.6 months [118] to about 14 months [120]. Remarkably, the studies reported by Tian et al. [119] and van der Kooij et al. [118] also included patients receiving pembrolizumab; however, the authors did not distinguish between both interventions when reporting the outcomes. In general, the patients tolerated the treatment well. Severe AEs were only reported for one patient who developed a grade 4 hyperglycemia due to ICB-related diabetes [120]. 

Altogether, in comparison with ipilimumab, patients receiving nivolumab achieved a similar median PFS but a longer median OS. The response rates varied from 0% up to a promising 25% in the retrospective analyses, whereas the ORR in the phase II trial was 5.8%, which is comparable to the results achieved with ipilimumab. However, the safety of nivolumab seems to be better than that of ipilimumab. 

#### 3.2.2. Pembrolizumab

The efficacy of pembrolizumab for the treatment of metastatic UM was evaluated in several expanded access programs (EAPs), retrospective analyses, and one phase II trial [115,121,122,123,124,125]. Kottschade et al. [121] and Karydis et al. [122] evaluated the efficacy of pembrolizumab in two EAPs including 10 and 25 patients, respectively. Kottschade et al. reported a high ORR of 37.5%; however, the number of enrolled and evaluated patients was relatively low. In contrast, Karydis et al. reported an ORR of only 8%. The median PFS were reported as 4 months (18 weeks) and 3 months (91 days), and the median OS was not reached in the study of Kottschade and not reported by Karydis. One patient developed severe AEs > grade 3. Recently, Johnson et al. published data on a small single-arm phase II study in five patients with metastatic UM (NCT02359851) [123]. One patient had a CR after receiving one dose, and two patients had a prolonged SD (20% response rate, 60% clinical benefit rate). However, these three patients had either no liver metastases or only small metastases. The median PFS was 11.0 months, which was almost three times longer than those achieved in the aforementioned EAPs. The median OS was not reached. Serious AEs > grade 3 were reported for one patient [123]. Rossi et al. [126] published the results of a prospective observational single-arm study evaluating the efficacy of pembrolizumab in 17 patients with metastatic UM. ORR was 11.7%, but no CR was achieved. The median PFS was 3.8 months, which is comparable to the results reported by Kottschade and Karydis [121,122]. Interestingly, patients with both liver and extrahepatic metastases had a longer median PFS than those with hepatic metastases only (8.4 vs. 3.1 months). No severe AEs > grade 3 were observed [126].

Additionally, three retrospective studies analyzed the efficacy of pembrolizumab and other anti-PD-1 and anti-PD-L1 antibodies [115,124,125]. In the publication of Bol et al. [115], the outcomes for each antibody were separately reported, whereas the other two publications did not clearly distinguish between the interventions. Bol et al. [115] compared the efficacy of pembrolizumab, ipilimumab, and a combined therapy with ipilimumab and nivolumab in a retrospective population-based study including 86 patients. ORR for pembrolizumab was 7%. The median PFS was 4.8 months and the median OS was 10.3 months [115]. Algazi et al. [124] published the results of a multicenter analysis including 56 metastatic UM patients who received either pembrolizumab, nivolumab or the anti-PD-L1 antibody atezolizumab. The median PFS was 2.6 months and the median OS was 7.7 months; however, the authors did not clearly distinguish between the interventions when reporting the outcomes. A PR was observed only in two patients who had received either nivolumab or pembrolizumab [124]. A second retrospective analysis published by Piperno-Neumann et al. [125] described the results of 21 metastatic UM patients who were treated with pembrolizumab or nivolumab. The median PFS was 3 months and thus similar to the one reported by Algazi et al. [125]. In general, the therapy was well tolerated by the patients as no serious AEs > grade 3 were reported in any of the three retrospective trials. 

In summary, PD-1 blocking antibodies showed a better clinical efficacy in patients with metastatic UM regarding the median OS and a more favorable toxicity profile compared to CTLA-4 blocking antibodies. The ORR achieved with nivolumab and pembrolizumab varied extremely in different trials, which makes it difficult to evaluate the efficacy.

### 3.3. Combination Therapies/Combined ICB: CTLA-4 and PD-1 Blockade

Several studies have evaluated the effect of combined ICB in UM. A combination of nivolumab and ipilimumab was assessed in an EAP reported by Shoushtari et al. [127]. Sixty-four patients with metastatic melanoma, including a UM subgroup of six patients, were included. No objective responses were achieved in the UM subgroup. The median PFS for UM patients was 2.8 months. No data on OS or serious AEs were specifically reported for the UM subgroup [127]. Piulats et al. reported data of an interim analysis of the prospective phase II GEM1402 trial (NCT02626962) [128]. The ORR was 12%, the median PFS was 3.3 months, and median OS was 12.7 months. A high number of patients (54%) developed grade 3 and 4 AEs, and nine patients discontinued the treatment [128]. 

Besides, four retrospective analyses investigated the efficacy of combined treatment approaches. One analysis published by Heppt et al. [129] compared the efficacy of PD-1 monotherapy using pembrolizumab or nivolumab with a combined anti-PD-1 and anti-CTLA-4 therapy in 101 UM patients. The median PFS were 3.1, 2.8, and 2.8 months and the median OS were 14 months, 10 months and not reached for pembrolizumab, nivolumab, and combined ICB, respectively. The ORRs were 3% and 5.7% for nivolumab and pembrolizumab monotherapy, respectively, in contrast to 16.7% for the combinational approach; however, no CR was observed in any treatment subgroup. Severe AEs ≥ grade 3 were more frequent in the treatment arm receiving the combination therapy compared to those receiving anti-PD-1 monotherapy (26.7% vs. 7.4% or 12.5%) [129]. Another multicenter analysis evaluated the efficacy of two ICB treatment combinations in 64 metastatic UM patients [130]. The patients received nivolumab or pembrolizumab plus ipilimumab. The authors did not distinguish between both combinations when reporting the results. The ORR was 15.6%, the median PFS was 3.0 months, and the median OS was estimated to be 16.1 months. Similar response rates were reported by Piulats et al. 2018 [128] and Heppt et al. 2017 [129] for combined ICB in this cancer entity as mentioned above. A high number of patients developed severe ICB-related AEs of grade 3 (24 patients, 37.5%) and grade 4 (1 patient, 1.6%) [130]. Furthermore, Karivedu et al. [131] published a retrospective analysis of eight metastatic UM patients who were treated with transarterial chemoembolization (TACE) and a combined ICB with nivolumab and ipilimumab. The median OS was 14 months and the median PFS was not reported. Two patients (25%) achieved a PR. Four patients developed treatment-related colitis; however, the severity of the AEs was not clearly reported. [131]. In a retrospective population-based study, Bol et al. [115] evaluated the efficacy of a combined therapy with ipilimumab and nivolumab in 19 patients. The combined approach led to an ORR of 21.1%. The median PFS was 3.7 months and the median OS was 18.9 months [115]. 

Taken together, the longest median OS was achieved with combined ICB. The median PFS was around 3 months and comparable among the different studies and antibodies. The longest median PFS of 9 months was achieved in a study investigating a treatment approach combining ICB, TAC, and systemic chemotherapy [114]. Throughout the studies, most of the responding patients achieved only a PR. Regarding safety, ipilimumab showed the most unfavorable toxicity profile as a high number of patients developed severe AEs > grade 3 but did not clinically benefit from this treatment. Thus, a combined ICB using PD-1- and CTLA-4-blocking antibodies seems to be the best option that is currently available for patients with metastatic UM. 

## 4. ICB in Uveal Melanoma—Doomed to Fail?

The enormous success of ICB for the treatment of metastatic CM raised the expectations that metastatic UM patients would also benefit from the new therapy options. However, as presented above, the results were rather disappointing. Several reasons might explain the poor response rates of ICB in metastatic UM as discussed in the following part.

### 4.1. Low Mutational Burden, Few Neoantigens, and Low PD-L1 Expression

Several studies have shown that ICB seems to be most effective in tumors with a high mutational burden, such as CM, cutaneous squamous cell carcinoma, or NSCLC [132,133], as these tumors are more likely to express a broad range of neoantigens that can be recognized by CTLs [134]. The exposure to environmental noxae, such as tobacco smoke, UV radiation, and other carcinogens facilitate the accumulation of dozens of mutations and result in the formation of tumors with a high mutational burden [12]. Currently, the tumor mutational burden is the best biomarker to predict response to ICB across tumor entities. In contrast to CM, UM is a tumor with a remarkably low mutational burden of around 0.5 per Mb sequence [20]. Environmental mutagens like UV radiation have been discussed as risk factors in UM; however, their impact on UM initiation and progression seems to be quite low and is still controversial [135]. Therefore, the probability of expressing neoantigens that are recognizable for T cells is relatively low. Additionally, the fact that only about 10% of UM primary tumors [99] and 5% of the UM cells in metastatic UM sites express PD-L1 [136] may also contribute to the low susceptibility of UM to ICB mediated by anti-PD-1 or anti-PD-L1 antibodies. Nevertheless, a small subset of patients appears to benefit from ICB, and durable responses have been achieved with ICB. Rodrigues et al. report the case of a UM patient with metastases in liver, lungs, and bones who had a CR after ICB with pembrolizumab [137]. Another UM patient, who had received ipilimumab plus pembrolizumab had an SD for 10 months and a prolonged survival of 2 years in the metastatic stage [138]. The tumors of both patients were characterized by an extraordinary high mutational burden and the presence of germline mutations of methyl-CpG-binding domain protein 4 (MBD4) [137,138] that are found in about 2% of UM patients [137]. Like the frequently in UM mutated BAP1, the *MBD4* gene is located on chromosome 3 (3q21.3) [139]. *MBD4* mutations are also present in up to 43% of colorectal, endometrial, gastric, and pancreatic cancer with microsatellite instability [139]. The protein acts as a DNA glycosylase and is involved in the base excision repair mechanism where it initiates the removal of deaminated cytosines and 5-methylcytosines and, in particular, thymine and uracil incorrectly pairing with guanine within CpG dinucleotides [140]. Altogether, these findings indicate that the low mutational burden of UM significantly contributes to the weak ICB susceptibility.

### 4.2. Development of the Primary Tumor in an Immune-Privileged Organ

UM arises in the eye in an immune-privileged environment that possesses inhibitory properties against both the innate and the adaptive immune system [141]. This may protect cancer cells not only at the site of the primary tumor but also may hamper a successful anti-tumor immune response in other sites of the body and thus contribute to ICB failure in UM. In contrast, autoimmune-mediated diseases such as uveitis can severely affect the eye. Nevertheless, several mechanisms in the eye, including physical barriers, the presence of immunosuppressive cells, and soluble as well as membrane-bound immune modulators, protect the sensitive retinal tissue from excessive immune responses that may cause vision loss and blindness [141]. The special anatomical structure of the eye limits the access of immune cells to the surrounding tissue through tight junctions between vascular endothelial cells forming the blood-ocular barrier and a lack of lymphatic vessels [142,143]. Additionally, soluble factors in the aqueous humor, including transforming growth factor-β (TGF-β), macrophage migration-inhibitory factor (MIF), vasointestinal polypeptide (VIP), and α-melanocyte-stimulating hormone (α-MSH) [144] dampen the immune response by inhibiting the IFN-γ secretion of activated CD4+ T cells [145]. TGF-β also inhibits the activity of proinflammatory macrophages [144] and upregulates CTLA-4 on CTLs, which promotes the formation of immunosuppressive T_regs_ [146]. T_regs_ are found in up to 24% of UM primary tumors, and their presence is associated with a poor prognosis [147]. T_regs_ formation is also stimulated by α-MSH, another soluble factor that is physiologically present in the aqueous humor [148], and can dampen neutrophil activation [142]. The soluble factor MIF is able to inhibit cell lysis mediated by natural killer (NK) cells [149]. As a part of the innate immune system, NK cells are able to recognize and destroy cells with a decreased or lacking MHC class I expression that would evade CTLs otherwise [150]. NK cells are able to destroy UM cells in vitro [151]. However, in contrast to most other tumors including CM [152], loss of MHC class I expression in UM is associated with a better prognosis [153,154].

UM cells not only evolve in an immunosuppressive environment, but they are also able to create or maintain this favorable environment also in other sites of the body by fostering mostly tumor-promoting immune cells and blocking tumor-killing T cells and NK cells. This is achieved by producing soluble mediators, such as TGF-β [155], α-MSH, and MIF [156], that are also present in the healthy eye. The expression of the membrane-bound PD-L1 was found in the retina, cornea, and iris [157,158,159], but also on UM cells [160]. However, unlike CM, only about 10% of UM primary tumors [99] and 5% of the UM cells in metastatic UM sites express PD-L1, but about 50% of tumor-infiltrating lymphocytes (TILs) express its receptor PD-1 [136]. Tumor-infiltrating immune cells in UM are mostly T cells and smaller numbers of NK cells, B cells, and macrophages [161]. Interestingly, a higher number of TILs has been associated with a worse prognosis in UM [162]. Macrophages in UM are mostly of the M2 phenotype, which facilitates angiogenesis and immunosuppression by producing anti-inflammatory cytokines such as TGF-β and IL-10, which dampen the activation and function of T cells, NK cells, and DCs [163]. Chemokines like CCL2 and CCL22, which are released by UM cells, promote the polarization towards this phenotype [164]. The presence of M2 macrophages in UM may also explain why a high number of TAMs in UM is also associated with a poorer prognosis [165].

### 4.3. Liver as an Immune-Modulating Organ

The liver is the primary organ where UM metastases develop. At time of death, hepatic metastases are present in more than 90% of UM patients [48]. Interestingly, the liver also acts as an immune-modulating organ. It is the first organ which the blood from the gastrointestinal tract passes and therefore needs a tightly balanced immune response in order to defeat pathogens like microbes and viruses quickly but maintain tolerance against harmless dietary antigens. This response is predominantly mediated via the components and resident cells of the innate immune system including the complement system and NK cells [166].

NK cells seem to play an important role in UM tumor growth and the establishment of hepatic metastases. Lymphocyte distribution in the liver significantly differs from other sites of the human body. About 30% to 50% of all lymphocytes in the liver are NK cells compared to 5% to 20% in the peripheral blood [167]. Disrupting the NK cell function in mouse models increased the number of UM liver metastases, whereas promoting NK cell function by interferon-β application resulted in a decreased formation of liver metastases [168,169]. These data indicate that UM cells are generally susceptible to NK-cell-mediated killing in the liver but may be protected in the immunosuppressive environment in the eye. Metastatic UM cells residing in the liver have developed several strategies to circumvent NK cell-mediated lysis. UM cell lines derived from hepatic metastases show a stronger production of MIF, a cytokine that inhibits NK cell function [156]. NK cells are activated via the natural-killer group 2 member D (NKG2D) receptor on their cell surface, which interacts with MHC class I related chain (MIC)-A/B molecules [170]. MIC-A/Bs are nonclassical MHC molecules and are expressed on the cell surface of various tumor entities, including about 50% of primary UMs [171]. Interestingly, it is absent in UM liver metastases, indicating that a decreased MIC-A/B expression also contributes to immune escape from NK-cell-mediated tumor cell lysis in the liver [171].

Besides this, the adaptive immune response against hepatic metastases is rather weak. Only small numbers of CTLs are found in UM liver metastases, and these are mostly observed peritumorally [172]. Thus, the infiltration of lymphocytes into the tissue is weak, and cell lysis mediated by CTLs seems to play a minor role in this process, indicating that ICB, which aims to boost the T cell-mediated anti-tumor response, is likely to fail. Besides this low accessibility to CTLs, only about 5% of the tumor cells in metastatic UM sites and 50% of TILs express PD-1 [136]. These observations are in line with several studies that showed poorer response rates of anti-PD-1 therapy also in CM with liver metastasis, also indicating that the T cell response in this organ plays only a minor role in tumor growth control [173,174]. Additionally, in the phase II study reported by Johnson et al. [123], the only patients who responded were those without or with only small hepatic metastases, and in the study presented by Rossi et al. [126], patients with liver metastases had a much lower median PFS compared to patients with extrahepatic metastases. 

### 4.4. Making Cold Tumors Hot for Immune Cells: Considerations for Future Immunotherapies

A special immune-modulating environment at the sites of the primary tumor, the liver as the preferred place for metastatic disease, and acquiring immunosuppressive properties help UM cells to evade from an anti-tumoral immune response. This partly explains why UM is less responsive to ICB than other cancer entities. However, despite the low number of neoantigens, UM expresses a couple of immunogenic antigens as melanoma antigen recognized by T cells (MART-1/Melan-A), glycoprotein 100 (gp100), tyrosinase, tyrosinase-related protein-1 (TRP-1) [175], or melanoma-associated antigen (MAGE) [176]. Furthermore, CTLs isolated from UM primary tumor tissue [177] or the peripheral blood of UM patients [178] as well as NK cells [151] are able to lyse UM cells in vitro. These findings demonstrate that other therapy options may be able to enhance an antitumoral immune response in UM despite the poor ICB results. Thus, is there a chance to develop an immunotherapy that can effectively mediate a successful antitumor response?

**Figure 2:**
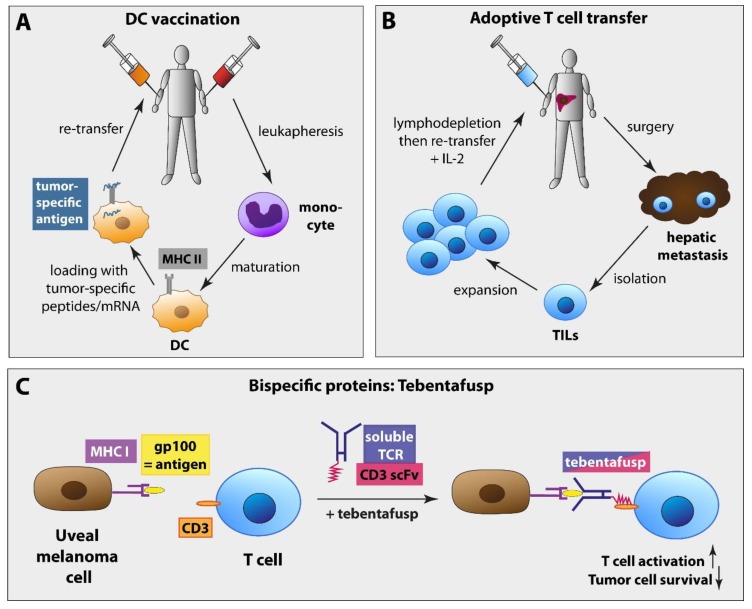
**Promising future immunotherapy options for UM patients**. (**A**) Dendritic cell vaccination: Monocytes or other hematopoietic progenitors are isolated from the tumor patient via leukapheresis and develop in vitro in the presence of stimulatory cytokines to mature DCs. These are then loaded with tumor-specific peptides (i.e., gp100, tyrosinase) or mRNA encoding these antigens and retransferred into the patient in order to boost the anti-tumor response. (**B**) Adoptive T cell transfer: TILs are isolated from hepatic metastases and expanded in vitro. After a lymphodepleting chemotherapy, the patient receives the expanded TILs followed by IL-2 administration. (**C**) Bispecific proteins, i.e., IMCgp100 (tebentafusp): The bispecific protein IMCgp100 combines a TCR against gp100 and CD3 scFv. The engineered TCR of the molecule binds to gp100 on UM cells presented by the MHC class I protein HLA-A*02:01, and the anti-CD3 antibody fragment binds to and activates CD3+ T cells. DC = dendritic cell, TILs: tumor-infiltrating lymphocytes, CD3 scFv = anti-CD3 single-chain antibody fragment, MHC I = major histocompatibility complex I, TCR = T cell receptor, IL-2 = interleukin-2.

Highly expressed antigens such as MART1, gp100, or TRP-1 may be suitable targets for, e.g., dendritic cell (DC) vaccination approaches. DCs are professional APCs and are required for the induction of an antigen-specific immune reaction by presenting captured antigens via MHC class I and II molecules to naïve CD8+ and CD4+ T cells [179]. Therapeutic DC vaccination uses this mechanism to provoke a specific antitumor immune response by generating tumor-specific CD8+ T cells and efficient CD4+ T helper cells. Monocytes or other hematopoietic progenitors are isolated from the tumor patient via leukapheresis and cultured in the presence of stimulatory cytokines such as IL-4 and granulocyte-macrophage colony-stimulating factor (GM-CSF) [179,180]. These monocyte-derived DCs are then loaded with tumor-specific peptides (i.e., gp100, tyrosinase) or mRNA encoding these antigens and retransferred into the patient [180] (Figure 2). In the last years, DC vaccination showed several promising results. A phase I/II trial investigating autologous DCs loaded with gp100 and tyrosinase in 14 metastatic UM patients achieved an antitumor immune response in four patients (29%), one SD, and a median OS with metastatic disease of 19.2 months. No serious AEs of grades 3 or 4 were reported, and the most frequently reported AEs were flu-like symptoms (eight patients, 57%), erythema at the site of the DC injection (six patients, 43%), and fatigue (five patients, 36%) [180]. Another open-label, nonrandomized phase II trial (NCT00929019) investigated DC vaccination in an adjuvant setting compared to observation only. Twenty-three UM patients with a high risk for developing metastases as characterized by monosomy 3 received autologous DCs loaded with tumor mRNA after resection of the primary tumor. DC vaccination achieved a 3-year OS of 79% and a median disease-free survival of 34.5 months. Similar to the study mentioned above, no grade 3 or 4 toxicities were observed, and the most common AEs were flu-like symptoms (21 patients, 91%) and erythema at the site of the DC injection (20 patients, 87%) [181]. Currently, a multicenter, open-label, randomized phase III trial investigating adjuvant DC vaccination versus observation only is ongoing (NCT01983748) [182]. Patients receive autologous DCs loaded with tumor mRNA intravenously over a period of 2 years or standard care with observation only and staging every three months.

The adoptive transfer of tumor-infiltrating lymphocytes (TILs) involves the isolation of TILs from hepatic metastases and their expansion in vitro. Before the expanded cells are retransferred, followed by a T-cell-stimulating IL-2 administration, the patients receive a lymphodepleting chemotherapy. This therapy option has shown promising results in a phase II trial (NCT01814046). Twenty-one HLA-A2-positive UM patients with hepatic metastases were included, and 20 patients were evaluable. One patient had a CR and six patients a PR (30%). Severe AEs of grade 3 and higher, including lymphopenia, neutropenia, and thrombocytopenia, were observed in all 21 patients, and 14 patients (67%) had a grade 3 anemia. These were most likely caused by the lymphodepleting chemotherapy but not by the adoptive T cell transfer itself. [151,183]. Currently, an interventional, open-label phase II trial (NCT03467516) investigating adoptive TIL transfer in metastatic UM patients has finished recruiting and will evaluate the ORR, CR rate, duration of response, disease control rate, PFS, and OS.

Another attempt includes IMCgp100 (tebentafusp), a bispecific protein that combines a TCR recognizing gp100 and an anti-CD3 single-chain antibody fragment (scFv). The engineered TCR binds to gp100 on UM cells presented by the MHC class I protein HLA-A*02:01 and the anti-CD3 antibody fragment binds to and activates CD3+ T cells [184]. IMCgp100 has achieved encouraging results in phase I/II trials. In a phase I first-in-human trial (NCT01211262) including 14 UM patients, two PR (14%), 8 SD (57%) and 4 PD (29%) were observed. [185]. Another phase I study (NCT02570308) investigated intra-patient escalating doses of IMCgp100 in 19 heavily pretreated metastatic UM patients. Two PR (11%) were observed in this trial, the median PFS was 5.6 months and the 1-year PFS was 62%, the 1-year OS 79.5% (95% CI 55–93), median OS was not yet reached. Rash (90%), pruritus (90%) and edema (63%) were the most frequently observed AEs. Similar to the first-in-human study, hypotension (16%) was the most frequent severe AE of grade 3 and higher [186]. Currently, a randomized controlled trial comparing IMCgp100 vs. physician’s choice chemotherapy or immunotherapy is recruiting patients with advanced UM (NCT03070392).

In highly prevalent tumor types, the use of high-throughput data (HTD) technologies for molecular profiling like DNA and RNA sequencing generates currently valuable knowledge to elucidate the molecular relationships behind pathogenesis, improve diagnostics, find druggable targets, and assess the mechanisms of resistance to therapy. The application of these technologies to UM is in its infancy [30]. However, in general, parallel and hierarchical integration analyses used for CM should also be applicable to other cancer entities as well [187]. These new techniques could significantly contribute to the understanding of UM pathogenesis and metastatic spread but also to the adaptation of therapies successful in other tumor entities like CM. As indicated, the liver is an immune-regulating organ which UM tumors benefit from developing. HTD techniques can be used to dissect the molecular features that make the liver microenvironment advantageous for UM. The use of single-cell RNA sequencing can provide detailed information about the populations of stromal and immune cells interacting in the tumor niche (see [188] for an example in CM). When combined with network biology methodologies, the RNA sequencing data can provide clues about the specific transcriptional programs that tumor and immune cells have activated [189,190]. The deep understanding of these programs, which govern key tumor and immune cell phenotypes, can help in finding molecular targets for coadjuvant therapy making UM cold tumors hot [191]. We also mentioned that UMs often have a low number of neoantigens, but still a number of overexpressed proteins holding immunogenic antigens. RNA sequencing analysis of tumor samples, differential expression, and computational algorithms for epitope detection can be combined to select in a personalized manner T cell epitopes from overly expressed proteins, which could be used in DC- or epitope-based immunotherapy [192].

## 5. Conclusions

UM is a rare malignancy and by far less susceptible to ICB than CM. Its genetic characteristics and the ability to adopt immunosuppressive strategies from the sites where it originates help UM cells to evade from the immune system and lead to checkpoint inhibitor resistance. However, some promising therapy approaches that aim to modulate and enhance the antitumoral immune response are in the pipeline and might prove successful in the next few years. As a subset of patients shows durable responses and clinical benefit to combined ICB, future studies are urgently warranted to better characterize and understand the underlying mechanisms of response.

## Figures and Tables

**Table 1:** Studies investigating immune checkpoint blockade for UM treatment.

Author/Trial [Reference]	Design	Number of Evaluated (Enrolled) Patients	Intervention	Dosage	ORR	PR	CR	PFS (Median)	OS (Median)	Severe AEs > grade 3
**Anti-CTLA-4 antibodies**										
**Ipilimumab**										
Rozeman 2019 SECIRA-UM [104]	Open-label, 3-armed, single center phase Ib/II trial	3	Ipilimumab + RFA	0.3 mg/kg	0	0	0	2 mo.	n.r.	1/3 (33%) *
		19	Ipilimumab + RFA	3 mg/kg	0	0	0	2 mo.	9.7 mo.	6/19 (32%) *
		19	Ipilimumab + RFA	10 mg/kg	0	0	0	2 mo.	14.2 mo.	10/19 (52%) *
Shaw 2012 [105]	EAP	18	Ipilimumab	3 mg/kg	n.r.	n.r.	n.r.	14.5 wks. (range 6 – 64)	n.r.	not clearly reported
Kelderman 2013/ WIN-O [106]	EAP	22	Ipilimumab	3 mg/kg	1/22 (4.5%)	1/22 (4.5%)	0	2.9 mo. (95% CI 2.3–5.3)	5.2 mo. (95% CI 4.9–9.6)	3/22 (13.6%)
Maio 2013 [107]	EAP	82 (83)	Ipilimumab	3 mg/kg	4/82 (4.8%)	4/82 (4.8%)	0	3.6 mo. (95% CI 2.8–4.4)	6.0 mo. (95% CI 4.3–7.7)	5/82 (6%)
Zimmer 2015 [108]	Observational, prospective, open-label, uncontrolled, multicenter phase II trial	53	Ipilimumab	3 mg/kg	0	0	0	2.8 mo. (95% CI 2.5–2.9)	6.8 mo. (95% CI 3.7–8.1)	19/53 (36%) *
Jung 2017 [109]	NPP	10	Ipilimumab	3 mg/kg	n.r.	n.r.	n.r.	2.8 mo.	not reached	0
Piulats 2014/ GEM1 [110]	Observational, prospective, open-label, single-arm phase II trial	31 (32)	Ipilimumab	10 mg/kg	2/31 (6.5%)	2/31 (6.5%)	0	n.r.	9.8 mo.	5/31 (16%)
Danielli 2012/ I-OMEAP [111]	EAP	13	Ipilimumab	10 mg/kg	0	0	0	n.r.	36 wks. (range 2–172+)	3/13 (23%)
Fountain 2019/ NCT01585194 [112]	Interventional, prospective, open-label, phase I/II trial	10	Ipilimumab (adjuvant setting)	3 mg/kg (*n* = 3) 10 mg/kg (*n* = 7)	n.r.	n.r.	n.r.	n.r.	n.r.	1/10 (10%)
Luke 2013 [113]	Uncontrolled, multicenter, retrospective analysis	39	Ipilimumab	3 mg/kg (*n* = 34) 10 mg/kg (*n* = 5)	2/39 (5.1%)	1 (late) (2.6%)	1/39 (2.6%)	n.r.	9.6 mo. (95% CI 6.3–13.4)	7/39 (17.9%)
Itchins 2017 [114]	Uncontrolled, single-center, retrospective cohort analysis	37	sequential TAC (fotemustine) + ICB (ipi, nivo or pembro) + systemic chemotherapy	100 mg/m² (fotemustine), 3 mg/kg (ipi/nivo) 2 mg/kg (pembro)	ICB first-line: 1/14 (7.1%) ICB second-line: 2/12 (16.7%)	Ipi: 1/22 (4.5%) Pembro: 2/15 (13%) Nivo: 1/4 (25%)	0	9 mo. (95% CI 6.2–13.2)	17.0 mo. (95% CI 12–26)	not clearly reported
**Tremelimumab**										
Joshua 2015 [116]	Observational, prospective, open-label, multicenter phase II study	11	Tremelimumab	15 mg/kg	0	0	0	2.9 mo. (95% CI 2.8–3.0)	12.8 mo. (95% CI 3.8–19.7)	not clearly reported
**Anti-PD-1 antibodies**										
**Nivolumab**										
Schadendorf 2017/ CheckMate172 [117]	Single-arm, open-label, multicenter, phase II trial	34 (75)	Nivolumab	3 mg/kg	2/34 (5.8%) at 12 wks.	2/34 (5.8%)	0	n.r.	11 mo. (95% CI 7–15)	not clearly reported
van der Kooij 2017 [118]	uncontrolled, multicenter, retrospective analysis	17	Nivolumab Pembrolizumab	3 mg/kg (nivo) 2 mg/kg (pembro)	0	0	0	2.3 mo.	9.6 mo.	0
Tian 2016 [119]	uncontrolled retrospective analysis	8 (9)	Nivolumab Pembrolizumab	n.r.	2/8 (25%)	2/8 (25%)	0	n.r.	not clearly reported	n.r.
Namikawa 2019 [120]	uncontrolled, single-center, retrospective analysis	14	Nivolumab	2 mg/kg (*n* = 13) 3 mg/kg (*n* = 1)	1/12 (7.1%)	1/12 (7.1%)	0	10 wks. (range 4–105)	60 wks. (range 5–105)	1/12 (7.1%) grade 4 hyper-glycemia
**Pembrolizumab**										
Kottschade 2016 [121]	EAP	8 (10)	Pembrolizumab	2 mg/kg	3/8 (37.5%)	2/8 (25%)	1/8(12.5%)	18 wks. (range 3.14–49.3)	n.r.	1/10 (10%)
Karydis 2016 [122]	EAP	25	Pembrolizumab	2 mg/kg	2/25 (8%)	2/25 (8%)	0	91 days	not reached	0
Johnson 2019/ NCT02359851 [123]	single-arm, multicenter, open-label, phase II trial	5	Pembrolizumab	n.r.	1/10 (20%)	0	1/5 (20%)	11.0 mo.	not reached	1/5 (20%)
Bol 2019 [115]	Retrospective, population-based study	43	Pembrolizumab	n.r.	3/43 (7%)	3/43 (7%)	0	4.8 mo.	10.3 mo.	n.r.
		24	Ipilimumab	n.r.	0	0	0	3.0 mo.	9.9 mo.	
		19	Ipilimumab + Nivolumab	n.r.	4/19 (21.1%)	4/19 (21.1%)	0	3.7 mo.	18.9 mo.	
Algazi 2016 [124]	uncontrolled, multicenter, retrospective analysis	38	Pembrolizumab	2 mg/kg (*n* = 27), 10 mg/kg (*n* = 9), unknown (*n* = 2)	2/56 (3.4%)	1/38 (2.6%)	0	2.6 mo. (95% CI 2.4–2.8)	7.7 mo. (95% CI 0.7–14.6)	0
		16	Nivolumab	1 mg/kg (*n* = 4), 2 mg/kg (*n* = 1), 3 mg/kg (*n* = 10), 10 mg/kg (n = 1)		1/16 (6.3%)	0			
		2	Atezolizumab	10 mg/kg (*n* = 1), 15 mg/kg (*n* = 1)		0	0			
Piperno-Neumann 2016 [125]	uncontrolled, single-center, retrospective analysis	21	Pembrolizumab Nivolumab	n.r.	0	0	0	3 mo.	n.r.	n.r.
Rossi 2019 [126]	Single-arm, prospective study	17	Pembrolizumab	2 mg/kg	2/17 (11.7%)	2/17 (11.7%)	0	3.8 mo. (95% CI 2.9–9.7)	not reached	0
**Combined ICB: anti-CTLA-4 + anti-PD-1 antibodies**										
Shoushtari 2016 [127]	EAP	6	Nivolumab + ipilimumab; nivolumab or pembrolizumab (maintenance)	1 mg/kg (nivo) + 3 mg/kg (ipi); 3 mg/kg (nivo, maintenance), 2 mg/kg (pembro, maintenance)	0	0	0	2.8 months (95% CI 1.2–4.6)	n.r.	n.r.
Piulats 2018/ GEM1402 NCT02626962 [128]	single-arm, open-label, multicenter, phase II trial	50 (52)	Nivolumab + ipilimumab; nivolumab (maintenance)	1 mg/kg (nivo) + 3 mg/kg (ipi); 3 mg/kg (nivo, maintenance)	6/50 (12%)	6/50 (12%)	0	3.3 mo.	12.7 mo.	not clearly reported
Heppt 2017 [129]	uncontrolled, multicenter, retrospective analysis	12 (15)	Nivolumab/ pembrolizumab + ipilimumab	3 mg/kg (ipi) + 1 mg/kg (nivo), 3 mg/kg (nivo, maintentance) (*n =* 7) 1 mg/kg (ipi) + 3 mg/kg (nivo), 3 mg/kg (nivo, maintentance) (*n* = 2) 1 mg/kg (ipi) + 2 mg/kg (pembro), 2 mg/kg (pembro, maintentance) (*n* = 6)	2/12 (16.7%)	2/12 (16.7%)	0	2.8 mo.	not reached	4/15 (26.7%) *
		53 (54)	Pembrolizumab monotherapy	2 mg/kg	3/53 (5.7%)	3/53 (5.7%)	0	3.1 mo.	14 mo.	4/54 (7.4%) *
		32	Nivolumab monotherapy	3 mg/kg	1/32 (3.1%)	1/32 (3.1%)	0	2.8 mo.	10 mo.	4/32 (12.5%) *
Heppt 2019 [130]	uncontrolled, multicenter retrospective analysis	59	Nivolumab + ipilimumab	3 mg/kg (ipi) + 1 mg/kg (nivo), 3 mg/kg (nivo, maintenance)	10/64 (15.6%)	8/64 (12.5%)	2/64 (3.1%)	3.0 mo. (95% CI 2.4–3.6)	16.1 months (95% CI 12.9–19.3)	1/64 (1.6%)
		5	Pembrolizumab + ipilimumab	1 mg/kg (ipi) + 2 mg/kg (pembro), 2 mg/kg (pembro, maintenance)						
Karivedu 2019 [131]	uncontrolled, single-center, retrospective analysis/case series	8	TACE + nivolumab + ipilimumab, TACE + nivolumab (maintenance)	3 mg/kg (ipi) + 1 mg/kg (nivo), 240 mg (nivo, maintenance)	2/8 (25%)	2/8 (25%)	0	n.r.	14 mo.	4/8 (50%) colitis; severity not clearly reported

All studies investigated ICB in patients with metastatic melanoma; exception: Fountain 2019/ NCT01585194 (adjuvant ICB in patients with a high risk for developing metastases). *: Authors did not distinguish between grade 3 and 4 when reporting the severity of AEs. Abbreviations: CI = confidence interval; DCR = disease control rate; EAP = expanded access program; ICB = immune checkpoint blockade; ipi = ipilimumab; mo. = months; nivo = nivolumab; NPP = named patient program; n.r. = not reported; pembro = pembrolizumab; RFA = radiofrequency ablation; TAC = transarterial chemotherapy; TACE = transarterial chemoembolization; wks. = weeks.

## Abbreviations

α-MSH	α-melanocyte-stimulating hormone
APCs	antigen-presenting cells
BAP1	BRCA1-associated protein 1
BRAF	v-Raf murine sarcoma viral oncogene homolog
CI	confidence interval
CM	cutaneous melanoma
CR	complete response
CTLA-4	cytotoxic T lymphocyte-associated antigen 4
CTLs	cytotoxic CD8+ T lymphocytes
CYSLTR2	G-protein coupled cysteinyl leukotriene receptor 2
DC	dendritic cell
DCR	disease control rate
EAP	expanded access program
EIF1AX	eukaryotic translation initiation factor 1A, x-linked
FDA	US Food and Drug Administration
GM-CSF	granulocyte-macrophage colony-stimulating factor
GNA11	guanine nucleotide-binding protein α11
GNAQ	guanine nucleotide-binding protein Q polypeptide
gp100	glycoprotein 100
HGF	hepatic growth factor
HLA	human leukocyte antigen
ICB	immune checkpoint blockade
IDO	indoleamine 2,3-dioxygenase
IFN-γ	interferon-γ
IGF-1	insulin-like growth factor-1
IL	Interleukin
MAGE	melanoma-associated antigen
MAPK	mitogen-activated protein kinase
MART-1/Melan-A	melanoma antigen recognized by T cells
MBD4	methyl-CpG-binding domain protein 4
MHC	major histocompatibility complex
MIC	MHC class I related chain
MIF	macrophage migration-inhibitory factor
NK cells	natural killer cells
NKG2D	natural-killer group 2 member D receptor
NRAS	neuroblastoma rat sarcoma viral oncogene homolog
NSCLC	nonsmall cell lung cancer
ORR	objective response rate
OS	overall survival
PD	progressive disease
PD-1	programmed death-1
PD-L1	programmed death ligand 1
PD-L2	programmed death ligand 2
PFS	progression-free survival
PI3K	phosphoinositide 3-kinase
PKC	protein kinase C
PLCB4	phospholipase C β4
PR	partial response
PTEN	phosphatase and tensin homolog
scFv	single-chain antibody fragment
SD	stable disease
SF3B1	splicing factor 3B, subunit 1
TAC	transarterial chemotherapy
TACE	transarterial chemoembolization
TAMs	tumor-associated macrophages
TCR	T cell receptor
TGF-β	transforming growth factor-β
TILs	tumor-infiltrating lymphocytes
TNF-α	tumor necrosis factor-α
T_regs_	regulatory T cells
TRP-1	tyrosinase-related protein-1
UM	uveal melanoma
UV	Ultraviolet
VCAM-1	vascular cell adhesion molecule-1
VIP	vasointestinal polypeptide
